# Emeritus unbound

**DOI:** 10.1038/s44319-026-00865-7

**Published:** 2026-07-07

**Authors:** L Trilling, D Chavalarias, J Ovadi, D Seebach, K Skarstad, G J White, V Norris

**Affiliations:** 1 Laboratoire TIMC (Recherche Translationnelle et Innovation en Médecine et Complexité), Université Grenoble Alpes, Bâtiment CReSI, La Tronche, France; 2 Institut des Systèmes Complexes Paris Île-de-France, Paris, France; 3 Institute of Molecular Life Sciences, HUN-REN Research Center for Natural Sciences, Budapest, Hungary; 4 ETH Zürich, Laboratorium für Organische Chemie, Zürich, Switzerland; 5 Department of Microbiology, Oslo University Hospital, Oslo, Norway; 6 Department of Physics and Astronomy, The Open University, Walton Hall, Milton Keynes, UK; 7 Laboratoire de Microbiologie CBSA (Communication Bactérienne et Stratégies Anti-infectieuses) - UR4312 Université de Rouen, Évreux, France

**Keywords:** Careers, Science Policy & Publishing

## Abstract

The contributions of emeritus professors to academia and to society in general are considerable but would be much greater if they were to receive adequate support. We suggest that ageism is one of the principal factors for the lack of support. We focus on science and give an example of how such support could be provided cheaply and efficiently.

**Figure:**
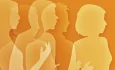

Emeritus professors make contributions to society that go well beyond what the title might suggest (Hirst and LeNavenec, 2022; Porter et al, 2026). In the case of scientists, on whom we focus here, emeritus professors continue to pursue research that often results in patents and publications. They also referee manuscripts and grant applications, mentor early-career colleagues, organise conferences, and advise governments and funding bodies. All this work draws on decades of accumulated experience and, notably, is provided at negligible or no cost to the institutions that benefit from it.

Notwithstanding, emeritus status is frequently treated as a temporary arrangement rather than a continuing relationship. When it lapses, retired scientists may find themselves without office space, without institutional email, and without the professional address through which collaborators have contacted them for years. Attendance at conferences becomes difficult to fund; open-access publication charges become a personal expense. While the scientific literature is increasingly accessible through preprint servers and open repositories, the loss of affiliation and professional networks is a more serious and less easily remedied problem. The cumulative effect can be damaging both to the individuals concerned (Kahana et al, 2018) and to the wider scientific enterprise that would benefit from their continued engagement. Allegedly, “An emeritus professor is like a eunuch in a harem: he knows how it is done, but he cannot do it anymore.”

The treatment of emeritus professors varies enormously across countries—and within them. This variation is itself telling, because it demonstrates that what emeriti can and cannot do after retirement is determined largely by institutional policy rather than by any inherent limitations. The USA offers a particularly permissive framework: NIH and NSF impose no age limits on grant eligibility, and formal federal emeritus programmes exist alongside visa provisions for foreign researchers. Within Europe, the picture is uneven. Sweden has no formal retirement age for professors; Leiden, KU Leuven, ETH Zürich, and EPFL all maintain active emeritus structures with continued access to laboratories and institutional resources. In France, Italy and Spain, by contrast, emeritus status tends to be honorary in practice, with little institutional support, which varies considerably between universities. In the UK, the default retirement age was abolished in 2011, but implementation has been inconsistent—older universities have generally been more accommodating than newer ones. That such different outcomes coexist within broadly comparable higher education systems is not a reflection of differing resources. It is a reflection of differing priorities.

One cannot help wondering whether some of these policies are shaped less by rational considerations than by other causes. One plausible candidate is ageism—a bias that is now widely normalised in contemporary culture. Modern societies tend to undervalue the contributions of older people in almost every domain, the political class being a notable exception. This is not merely speculative in an academic context: analysis of responses to a proposed NIH Emeritus Grant mechanism found that many respondents expressed strongly negative attitudes towards continued funding of older investigators, with many advocating for forced retirement—attitudes that the authors characterised as reflecting intergenerational conflict and social closure rather than evidence-based assessment of scientific productivity (Kahana et al, 2018). That such attitudes are widespread beyond academia is well-documented (Chang et al, 2020). Both run counter to commitments to reduce age discrimination and promote the employment of older workers—but sit comfortably alongside market-driven assumptions in which productive value is equated with youth and those past a certain age are regarded as spent.

When applied to scientists, the evidence does not support a picture of sharp intellectual decline at retirement age. Data from the 2023 NSF Survey of Doctorate Recipients show that nearly 40% of US-trained doctoral scientists and engineers aged 71–75 remained in active employment (Chang et al, 2020). None of this establishes that all emeriti remain productive—of course they do not—but it does undermine any categorical claim that retirement age marks a natural terminus.

Reliable, specific data on emeritus productivity are, admittedly, hard to come by. Publication rates, citation trajectories and funding outcomes for retired scientists are rarely tracked in a systematic way, and this gap in the record is itself a problem. Without it, the debate about what emeriti contribute is too easily driven by anecdote, which can be selected to support whichever position. What can be said with confidence is that productivity is highly individual at every age, and that the question worth asking is not whether retired scientists are, on average, still contributing, but whether the systems we have in place provide those who wish to continue and contribute to science with the means to do so.

The most common objection to continued support for emeritus researchers is that it competes with funding for early-career scientists, who already face an extremely difficult situation of short-term contracts, limited positions and intense competition for a shrinking pool of resources. This concern is real and should be taken seriously. But the assumption that the two groups are inevitably in competition rests on a zero-sum view of research funding that is questionable on both empirical and practical grounds. Emeriti do not generally need the same kind of support as early-career researchers; they are more likely to bring in collaborative funding, contribute to ongoing projects, and take on the reviewing and administrative work that otherwise falls disproportionately on mid-career colleagues. The question is not whether a fixed pot of money should go to older or younger scientists. It is whether the research system as a whole benefits from keeping experienced scientists connected to it.

There are at least four practical reasons for funding emeritus professors. First, they take on a share of refereeing, reviewing and committee work that would otherwise burden younger colleagues at precisely the stage when they need time for research. Second, they continue to make discoveries—sometimes significant ones. Third, their international networks and experience represent valuable assets for younger colleagues. Fourth, the cost of keeping them engaged is, by any measure, small. That said, it should be acknowledged that not all emeriti are assets. Those who have dominated a field for decades may inadvertently inhibit new approaches. This is not a trivial concern, and our proposals do not ignore it. Continued institutional affiliation could reasonably be made subject to periodic light-touch review—not of productivity in the narrow sense, but active engagement with the research community. Time-limited renewable arrangements offer a practical model: they preserve the connection while creating natural points at which both parties can reassess whether it remains mutually beneficial.

How could a better system work? We propose four practical steps.

The first is the simplest: institutions should be encouraged to provide emeritus professors with the basic infrastructure they need to remain active: institutional affiliation, email, library access and, where feasible, workspace. The cost is negligible, but the signal it sends about the value placed on their scientific experience is not.

Second, international mobility should be encouraged. Irrespective of their age, foreign scientists are encouraged to visit the USA, and retired scientists could be encouraged to move between European countries. They would undertake missions from one country to another, rather than contributing to a brain drain to countries with more flexible policies. Their contributions—publications, patents, training, start-ups—would provide natural criteria for evaluating whether the arrangement was working.

Third, funding bodies should adopt US policies that grants are open to scientists of any age, assessed on merit alone, provided the applicant holds an institutional affiliation sufficient to serve as Principal Investigator. Where specific emeritus funding schemes are created, they should be designed explicitly to complement rather than compete with early-career provision—through collaborative projects that pair senior expertise with junior innovation, or through synthesis work that draws on extensive experience or well-established networks.

Fourth, they should receive modest funding that would allow them to advance their individual or collaborative research projects—for instance, by paying for a postdoc at the end of their grant. Again, the outcome in terms of publications, patents and start-ups would serve as criteria for evaluation.

The financial case for action is straightforward since the costs are modest when compared with major research funding programmes. For example, supporting a thousand emeritus professors with funding of up to ten thousand euros each would cost ten million euros per year—a figure that is invisible against the €93.5 billion budget of Horizon Europe 2021–2027. The human case is perhaps more important: science is conducted by people, and the knowledge, judgement and connections that accumulate over a long career do not expire when the employment ends. A research system that discards these resources because of the age of the person holding them is not being efficient. It is being wasteful.

## Supplementary information

Peer Review File

## References

[CR1] Chang E-S, Kannoth S, Levy S, Wang S-Y, Lee JE, Levy BR (2020) Global reach of ageism on older persons’ health: a systematic review. PLoS ONE 15(1):e0220857 31940338 10.1371/journal.pone.0220857 PMC6961830

[CR2] Hirst S, LeNavenec C-L (2022) Professor emeritus: a “neglected” mentor on university campuses. In: Papers on postsecondary learning and teaching. University of Calgary (eds. Jeffs, C. & Fedorko-Bartos, K.-M.) Vol. 5, pp 100–110

[CR3] Kahana E, Slone MR, Kahana B, Langendoerfer KB, Reynolds C (2018) Beyond ageist attitudes: researchers call for NIH action to limit funding for older academics. Gerontologist 58:251–260 28073997 10.1093/geront/gnw190 PMC5946956

[CR4] Porter MM, Kops W, Kauenhowen C (2026) Experiences of Senior Scholars and Professors Emeriti—a mixed methods study. J High Educ Policy Manag 48:1–16. 10.1080/1360080X.2025.2498709

