# Author Correction: Emeritus unbound

**DOI:** 10.1038/s44319-026-00889-z

**Published:** 2026-07-30

**Authors:** L Trilling, D Chavalarias, J Ovadi, D Seebach, K Skarstad, G J White, V Norris

**Affiliations:** 1 Laboratoire TIMC (Recherche Translationnelle et Innovation en Médecine et Complexité), Université Grenoble Alpes, Bâtiment CReSI, La Tronche, France; 2 Institut des Systèmes Complexes Paris Île-de-France, Paris, France; 3 Institute of Molecular Life Sciences, HUN-REN Research Center for Natural Sciences, Budapest, Hungary; 4 ETH Zürich, Laboratorium für Organische Chemie, Zürich, Switzerland; 5 Department of Microbiology, Oslo University Hospital, Oslo, Norway; 6 Department of Physics and Astronomy, The Open University, Walton Hall, Milton Keynes, UK; 7 Laboratoire de Microbiologie CBSA (Communication Bactérienne et Stratégies Anti-infectieuses) - UR4312 Université de Rouen, Évreux, France

## Abstract

**Figure:**
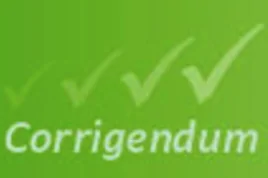

**Correction to:** *EMBO Reports* (2026) XX:XX. https://doi.org/10.1038/s44319-026-00865-7 | Published online 07 July 2026

In this article, the author’s name ‘D Seebach’ was incorrectly written as ‘G D Seebach’. The original article has been corrected.

