# Supplementary material for: Emeritus unbound
Source: EMBO Rep. 2026 Jul 7;27(17):4937–9. doi: 10.1038/s44319-026-00865-7 (PMC13554126; doi:10.1038/s44319-026-00865-7)
Supplement: Supplementary file 1 [file 44319_2026_865_MOESM1_ESM.pdf]

EMBO reports

Emeritus Unbound

Laurent Trilling, David Chavalarias, Judit Ovadi, Dieter Seebach, Kirsten Skarstad, Glenn White, and Vic Norris

Corresponding author(s): Vic Norris (victor.norris@univ-rouen.fr)

Review Timeline:

Submission Date:	1st Jun 26
Editorial Decision:	5th Jun 26
Revision Received:	9th Jun 26
Accepted:	30th Jun 26

Editor: Holger Breithaupt

Transaction Report: This article type is not peer reviewed.
